# 

**Published:** 1895-12-14

**Authors:** 


### CONTENTS.

**Is a Small-pox Hospital a Nuisance?** - 175

**Can the Treatment of the Insane be Improved?** - 176

**The Treatment of Epilepsy** - 177

**Heredity and the Race** - 178

**Notes and News** - 180

**Medical Progress and Hospital Clinics—**

The Case for Vaccination—From a Pathological Standpoint 181

Progress in Medicine.—General Pathology - 182

Progress in Surgery.—General Surgery - 183

Genito-Urinary Surgery - 185

New Appliances and Things Medical - 186

**Annotations.**—How to Get into a Fever Hospital—A Sanitary Record for Hull—The Clinics of the Future - 179

**The Institutional Workshop—**

Hospital Expenditure.—The Commissariat. IX.—The Nurses' Table—Provincial - 187

The "Coromandel"—The Hospital Ship for the Ashanti Expedition - 188

Practical Departments. III.—Furniture and Fittings for Nurses' Homes - 188

The Story of the Insane from Year to Year - 189

Around the Hospitals - 190

**Scraps and Gleanings** - 190

### EXTRA SUPPLEMENT.—THE NURSING MIRROR.

**News from the Nursing World.**—Our Needlework Competition—Nurses at Westminster Hospital—Miss Nightingale's Opinion of Registration—Nursing Sisters for Ashantee—Universal Uniform—The Nurse as Clinical Clerk—A Plea for Old Customs—Workhouse Infirmary Nursing Association—Badges at Salisbury—Night and Day Duty—Prince Alfred Hospital, Sydney—Short Items lxxxii

**Elementary Physiology for Nurses.** By C. F. Marshall, M.D., F.R.C.S. XIX.—The Eye (*Concluded*) - lxxxv

**What Christmas Means to Hospital Officials** - lxxxvi

**Miss Nightingale in Buckinghamshire** - lxxxvii

**Where to Go** - lxxxvii

**The Princess Maud Marriage Present Fund** - lxxxviii

**Help the Nurses to Help the Sick** - lxxxix

**Christmas and New Year Gifts** - xc

**Appointments** - xc

**Dundee Royal Lunatic Asylum** - lxxxviii

**Royal British Nurses' Association** - xci

**Christmas Books** - xcii

**Books for the Young** - xcii

**Notes and Queries** - xcii

**Everybody's Opinion** - xciv

